# Association between underweight and tooth loss among Korean adults

**DOI:** 10.1038/srep41524

**Published:** 2017-01-27

**Authors:** In-Seok Song, Kyungdo Han, Jae-Jun Ryu, Jun-Beom Park

**Affiliations:** 1 Department of Oral and Maxillofacial Surgery, Korea University Anam Hospital, Seoul, Republic of Korea; 2 Department of Biostatistics, College of Medicine, The Catholic University of Korea, Seoul, Republic of Korea; 3 Department of Prosthodontics, Korea University Anam Hospital, Seoul, Republic of Korea; 4 Department of Periodontics, College of Medicine, The Catholic University of Korea, Seoul, Republic of Korea

## Abstract

There is growing interest in the relationship between body mass index and oral health. Previous study showed that being underweight was significantly associated with having lower masticatory performance. This study was performed to assess the relationship between an underweight body mass index lower than 18.5 and the number of natural teeth using nationally representative data. Initially, a total of 25,534 individuals were candidates in the Korean National Health and Nutrition Examination Survey. The analysis in this study was confined to 17,870 subjects who were 19 years or older and without missing values for outcome variables. Body mass index and number of natural teeth were evaluated. Multiple regression analysis was used to evaluate the risk of tooth loss in relation to body mass index. Adjusted odds ratios and their 95% confidence intervals for chewing discomfort in individuals who were underweight, normal, overweight, obese, and extremely obese were 1.712(1.156–2.535), 1.111(0.939–1.315), 1(reference), 0.949(0.798–1.128), and 1.172(0.807–1.700), respectively, after adjustment. The association between underweight and tooth loss was proven by multiple logistic regression analyses after adjusting for confounding factors. Underweight may be considered a potential risk indicator for tooth loss in Korean adults.

There is growing interest in the relationship between body mass index and oral health because both are significant public health concerns^1^. Body mass index has been reported to be related to periodontitis, suggesting that obesity is a risk factor for periodontitis^2^. Similarly, being overweight has been considered a risk factor for periodontitis^3^. Overweight and obese preschool children have been shown to be at higher risk for dental caries than normal- and underweight children^4^. Conversely, in a multivariate logistic regression analysis, no association was found between body mass index and periodontitis^5^. In a previous study, it was shown that body mass index was not related to dental caries^2^. A sex difference was reported in a previous report, showing that obesity appeared to be a risk factor for periodontal attachment loss progression for females but not males^6^.

As for underweight, relatively fewer studies have been performed regarding the relationship with oral health^7^,^8^,^9^. The percentage of participants who were underweight varied depending on the studies—4.8%^2^, 7.74%^1^, and 13.6%^10^. Underweight has been reported to be associated with a higher risk of all-cause mortality in chronic obstructive pulmonary disease and cardiovascular disease^11^,^12^. Furthermore, being underweight has been reported to raise the risk of fracture and bone loss^13^. In multiple logistic regression analyses, it was shown that being underweight was significantly associated with having lower masticatory performance (odds ratio of 2.0)^7^. In addition, people without teeth were significantly more likely to be underweight than those with 11 or more teeth^9^. It was hypothesized that there is no significant association between underweight and number of natural teeth. Thus, this study was performed to assess the relationship between underweight, with a body mass index lower than 18.5, and number of natural teeth using nationally representative data.

## Materials and Methods

### Subjects

The data for this study were obtained from the Korean National Health and Nutrition Examination Survey (KNHANES) in 2010–2012. This survey is conducted by the Korean Ministry of Health and Welfare annually to monitor the general health and nutritional status of the Republic of Korea population^14^. The KNHANES is composed of a health examination survey, a health interview survey, and a nutritional survey by trained staff members. A rolling sampling design that involves complex, stratified, and multistage probability samples is used to collect the data. The KNHANES was approved by the Institutional Review Board of the Korea Centers for Disease Control, and all participants signed an informed consent form. This study was conducted according to the Helsinki Declaration-based ethical principles for medical research involving human subjects.

**Figure 1:**
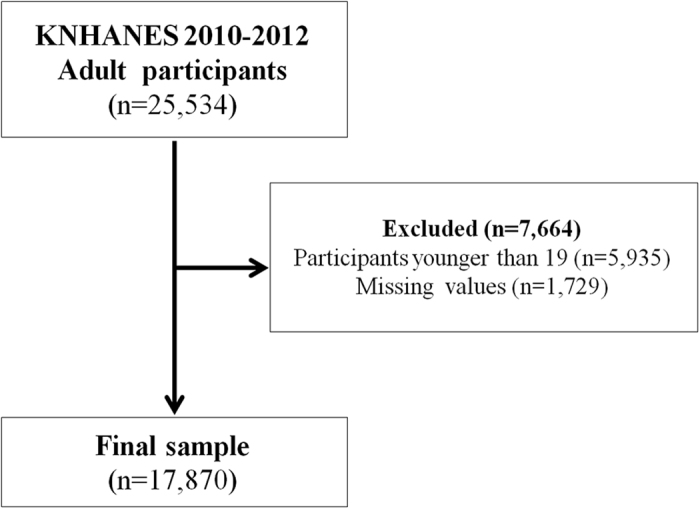
Participant flow chart.

Initially, a total of 25,534 individuals were candidates in the KNHANES survey. The analysis in this study was confined to a total of 19,599 respondents over 19 years old. Finally, 17,870 individuals without missing values for the outcome variables were analyzed for the analysis (Fig. 1).

### Measurement and classification of variables

Anthropometric measurements were performed by trained staff members. Body weight and height were measured with the subject wearing light clothing, and body mass index was calculated using the following formula: body mass index = weight (kg)/height (m^2^). Waist circumference was measured at the level midway between the costal margin and the iliac crest at the end of a normal expiration.

Smoking status was categorized as current smoker or not from the interview. Individuals were categorized using the criterion for alcohol consumed within one month in accordance with respondents’ answers on the self-reported questionnaire^15^. Individuals were regarded as regular physical exercisers if they performed moderate exercise at least 5 times per week for at least 30 minutes per session or performed vigorous exercise at least 3 times per week for at least 20 minutes per session^16^. Socioeconomic status was indicated by monthly household income and education level. Monthly household income level was divided into quartiles; the lowest quartile included households with a monthly income <$1092.4. Education level was categorized as high school graduate or higher. Residential areas were categorized into urban areas in the case of administrative divisions of “dong”^17^. Having a spouse and recognition of stress were self-reported.

Concentrations of serum fasting plasma glucose, total cholesterol, triglycerides, and high-density lipoprotein cholesterol were measured from blood samples collected from the antecubital vein after fasting for more than eight hours. Metabolic syndrome was defined according to the American Heart Association/National Heart, Lung, and Blood Institute Scientific Statement criteria for Asians^18^. To be diagnosed with metabolic syndrome, three or more of the following criteria must be fulfilled: fasting triglycerides ≥150 mg/dL or use of lipid-lowering medication; high-density lipoprotein cholesterol <40 mg/dL in men and <50 mg/dL in women or use of cholesterol-lowering medication; waist circumference ≥90 cm in men and ≥80 cm in women; blood pressure ≥130/85 mm Hg or use of antihypertensive medication; or fasting blood glucose ≥100 mg/dL or current use of antidiabetic medication. Diabetes was diagnosed when fasting blood sugar was >126 mg/dL or when the individual was currently using antidiabetic medications^19^. Hypertension was defined as a systolic blood pressure of >160 mm Hg, a diastolic blood pressure of >90 mm Hg, or the current use of systemic antihypertensive drugs^20^. The level of kidney function was determined by estimated glomerular filtration rate (eGFR) using the following equation: eGFR (mL/min/1.73 m^2^) = 186.3 × (serum creatinine^−1.154^) × (age^−0.203^); this result was then multiplied by the constant 0.742 if the patient was female^21^,^22^. Stroke was defined if diagnosed by a doctor or if the individual had experienced complications. Congenital heart defect was defined if diagnosed by a doctor. Cardiovascular disease was considered present if the individual had experienced stroke and congenital heart defect^23^. Hypercholesterolemia was considered as a fasting blood cholesterol level >240 mg/mL or use of medication for the condition^24^.

### Oral health behaviors and number of natural teeth

In this study, the time of day when toothbrushing was undertaken was evaluated. We calculated the frequency of daily toothbrushing by the total number of times participants brushed their teeth per day^25^. Dental checkup within a year, experience of dental pain within a year, and self-reported oral status were also evaluated.

### Statistical analysis

The data are presented as means ± standard errors for continuous variables and as proportions (standard errors) for categorical variables. A chi-square test for categorical variables or an independent t-test for continuous variables was performed to assess the differences in characteristics categorized by body mass index. Multiple regression analysis was used to evaluate the risk of tooth loss in relation to body mass index. The odds ratios and 95% confidence intervals were calculated to identify the risk of tooth loss. Model was adjusted for age, sex, smoking, drinking, exercise, income, education, metabolic syndrome, stress, and frequency of toothbrushing.

## Results

Table 1 describes the baseline characteristics of the study individuals according to body mass index. Age was significantly lower in participants with a body mass index lower than 18.5. The percentage of current smokers and current drinkers was significantly lower in underweight participants. The percentage of individuals with a high school or higher education was higher in the underweight group. The percentage of metabolic syndrome was significantly lower in underweight participants.

**Figure 2:**
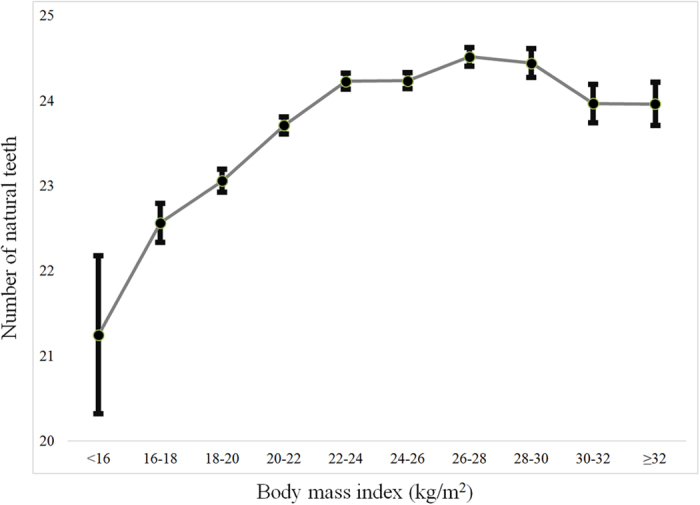
Average number of natural teeth categorized by body mass index.

Figure 2 shows the average number of natural teeth categorized by body mass index. The average number of natural teeth showed increasing trends up to a body mass index of 26 to 28 (*P* < 0.05).

Table 2 shows the association between number of natural teeth and oral health behavior in systemic diseases. Cardiovascular disease, stroke, congenital heart defect, diabetes mellitus, hypertension, hypercholesterolemia, metabolic syndrome, body mass index, and waist circumference were significantly associated with the number of natural teeth (*P* < 0.05).

Table 3 indicates the average number of natural teeth categorized by body mass index. In the adjusted model, the number of natural teeth was lowest in the underweight group with a body mass index lower than 18.5 (*P* < 0.05).

Table 4 shows the adjusted odds ratios and their 95% confidence intervals from multivariate logistic regression analyses for individuals with less than 20 natural teeth categorized by body mass index after adjustment for age, sex, smoking, drinking, exercise, income, education, metabolic syndrome, stress, and frequency of toothbrushing. Adjusted odds ratios and their 95% confidence intervals with less than 20 natural teeth in individuals who were underweight, normal, overweight, obese, and extremely obese were 1.712(1.156–2.535), 1.111(0.939–1.315), 1(reference), 0.949(0.798–1.128), and 1.172(0.807–1.700), respectively, after adjustment.

## Discussion

This study aimed to identify associations between number of natural teeth and body mass index using nationally representative data. The results showed that an increased risk of tooth loss was associated, with statistically significant differences, with participants who were underweight.

This association may be explained by the following. The participants who were underweight have the possibility of undernutrition^26^. Being underweight may mean the participants are deficient in a healthy, balanced diet^27^. Underweight individuals may have inadequate intake and absorption of essential amino acids and vitamins^28^,^29^. Underweight individuals may be prone to infectious disease due to weaker immune systems^30^. Poor masticatory performance also has a significant relationship with being underweight^8^. For example, it was reported that participants who were underweight chewed more asymmetrically and more slowly than normal-weight or obese participants^31^. Furthermore, children with at least one decayed tooth were reported to be significantly underweight, with odds ratios of 1.6 (95% confidence interval: 1.1–2.3) and 1.5 (95% confidence interval: 1.1–2.0) for 6–8-year-old and 9–12-year-old children, respectively, in the adjusted model^32^. Children with severe early childhood caries had a weight below the 3rd percentile (underweight; mean 15.49 ± 1.87 kg), which was less than the controls (mean weight of 16.34 ± 1.46 kg)^33^.

The present study identified that underweight can be considered a risk factor for tooth loss among Korean adults. However, previous reports have shown conflicting results. Similar to our results, postmenopausal women who were underweight showed increased risk of tooth loss, especially of the anterior teeth, compared with women who had a normal body mass index^34^. Edentulous participants were more likely to be underfat, with odds ratios of 3.11 (95% confidence interval: 1.27–7.61)^35^. Conversely, 44.7% of underweight children showed natural, healthy teeth, as did 40.7% of those with normal weight, whereas only 30.5% and 31.7% of children with a high weight and obesity, respectively, showed natural, healthy teeth^36^. However, a previous report showed that there were no significant differences in the number of remaining teeth between under- and overweight participants^34^. In another report, the authors did not observe large differences in masticatory performance or swallowing variables among the four different weight groups^37^.

Various methods have been used to assess obesity, including body mass index, waist circumference, and percentage of body fat^5^. Another report measured the circumferences of the waist and the hip in order to provide the waist–hip ratio^38^. In a previous report, no association was found between body mass index and periodontitis^5^. However, a high waist circumference seems to be associated with periodontitis, suggesting that abdominal obesity is significantly correlated with periodontitis^5^. Central obesity is more prevalent than general obesity among older adults^39^, and visceral fat area has been suggested to be the most suitable indicator of obesity in relation to periodontitis^40^.

This study had several limitations. Due to the cross-sectional design of the study, the causal direction of the associations cannot be ascertained^41^. A longitudinal study may prove a cause-and-effect relationship regarding underweight and tooth loss. Additionally, in this study underweight was defined using body mass index, but other methods may present an individual’s status better. However, it should be stressed that body mass index is one of the most widely used methods for evaluating weight status. The strengths of the data lie here: The number of natural teeth was used as a representation of oral health status, and it is one of the most efficient and reliable methods^42^. In addition, the data used in this study were obtained by a rolling sampling design that involved complex, stratified, and multistage probability samples, and therefore, these data can be considered nationally representative^43^,^44^. The association between number of natural teeth and body mass index was proven by multiple logistic regression analyses after adjusting for confounding factors, and consequently, the results can be considered representative and reliable.

In conclusion, the association between underweight and tooth loss was proven by multiple logistic regression analyses after adjusting for confounding factors. Underweight may be considered a potential risk indicator for tooth loss in Korean adults.

## Additional Information

**How to cite this article**: Song, I.-S. *et al*. Association between underweight and tooth loss among Korean adults. *Sci. Rep.* **7**, 41524; doi: 10.1038/srep41524 (2017).

**Publisher's note:** Springer Nature remains neutral with regard to jurisdictional claims in published maps and institutional affiliations.

## Figures and Tables

**Table 1:** Baseline characteristics of study participants according to body mass index lower than 18.5.

Unweighted n	Body mass index <18.5 kg/m^2^		
	No	Yes	*P-*value^*^
	17043	827	
Number of natural teeth	24.6 ± 0.1	24.6 ± 0.3	0.9276
Age (years)	46.0 ± 0.2	38.0 ± 0.8	<0.0001
Sex (male)	50.3 (0.4)	28.6 (2.1)	<0.0001
Smoking (current)	23.9 (0.5)	19.6 (1.9)	0.0423
Drinking (current)	58.6 (0.6)	53.7 (2.3)	0.0369
Exercise (yes)	20.0 (0.5)	11.7 (1.4)	<0.0001
Income (lowest quartile)	16.3 (0.5)	18.0 (1.8)	0.3293
High school graduate or higher	70.4 (0.7)	81.3(1.7)	<0.0001
Cardiovascular disease (yes)	2.7 (0.1)	1.1 (0.3)	0.0024
Stroke (yes)	0.8 (0.1)	0.4 (0.2)	0.0722
Congenital heart defect (yes)	2.0 (0.1)	0.8 (0.3)	0.0164
Diabetes mellitus (yes)	8.7 (0.3)	2.2 (0.5)	<0.0001
Hypertension (yes)	28.4 (0.5)	9.6 (1.1)	<0.0001
Hypercholesterolemia (yes)	12.9 (0.3)	2.3 (0.6)	<0.0001
Chronic kidney disese (Estimated glomerular filtration rate <60 mL/min/1.73 m)	1.9 (0.1)	1.5 (0.4)	0.4532
Metabolic syndrome (yes)	27.2 (0.5)	2.4 (0.5)	<0.0001
Stress (yes)	27.5 (0.4)	32.0 (2.2)	0.0339
Frequency of toothbrushing per day			0.0032
≤1	12.0 (0.4)	9.8 (1.2)	
2	45.4 (0.6)	39.9 (2.4)	
≥3	42.7 (0.7)	50.3 (2.4)	
Body mass index category			
Underweight (x < 18.5 kg/m^2^)	0.0 (0.0)	100.0 (0.0)	
Normal (18.5 ≤ x < 23 kg/m^2^)	42.4 (0.5)	0.0 (0.0)	
Overweight (23 ≤ x < 25 kg/m^2^)	23.9 (0.4)	0.0 (0.0)	
Obese (25 ≤ x < 30 kg/m^2^)	29.0 (0.4)	0.0 (0.0)	
Extreme obese (x ≥ 30 kg/m^2^)	4.7 (0.2)	0.0 (0.0)	

Data are presented as means ± standard error or percentages (standard error).

^*^P-values were obtained by independent t-test for continuous variables or chi-square test for categorical variables.

**Table 2:** The association between number of natural teeth and oral health behavior in systemic diseases.

n	Number of natural teeth			
	≤20	21–27	28	*P-*value
	3,475	7,342	7,053	
Cardiovascular disease (yes)	8.3 (0.6)	2.9 (0.2)	0.8 (0.1)	<0.0001
Stroke (yes)	2.5 (0.3)	0.8 (0.1)	0.2 (0.1)	<0.0001
Congenital heart defect (yes)	6.0 (0.5)	2.2 (0.2)	0.6 (0.1)	<0.0001
Diabetes mellitus (yes)	20.8 (0.9)	10 (0.5)	3.9 (0.3)	<0.0001
Hypertension (yes)	56.0 (1.1)	32.2 (0.7)	15.5 (0.5)	<0.0001
Hypercholesterolemia (yes)	20.0 (1.0)	14.7 (0.5)	8.6 (0.4)	<0.0001
Chronic kidney disease (Estimated glomerular filtration rate <60 mL/min/1.73 m)	7.8 (0.6)	1.8 (0.2)	0.4 (0.1)	<0.0001
Metabolic syndrome (yes)	48.9 (1.2)	30.1 (0.7)	16.6 (0.6)	<0.0001
Normal to extreme obese individuals	32.9 (1.1)	34.4 (0.7)	29.7 (0.7)	<0.0001

Data are presented as percentages (standard error).

^*^P-values were obtained by chi-square test for categorical variables.

**Table 3:** The number of natural teeth categorized by body mass index.

Body mass index	Model
<18.5 (underweight)	22.6 ± 0.2
18.5 ≤ x < 23 (normal)	23.7 ± 0.1
23 ≤ x < 25 (overweight)	24.3 ± 0.1
25 ≤ x < 30 (obesity)	24.4 ± 0.1
≥30 (extreme obesity)	24.0 ± 0.2
*P*-value	<0.0001

Data are presented as means ± standard error.

Model: age, sex, smoking, drinking, exercise, income, education, metabolic syndrome, stress, and frequency of toothbrushing adjusted.

**Table 4:** Adjusted odds ratios and 95% confidence intervals of individuals with less than 20 natural teeth categorized by body mass index in multivariate logistic regression models.

Body mass index	Model
<18.5 (underweight)	1.712 (1.156–2.535)
18.5 ≤ x < 23 (normal)	1.111 (0.939–1.315)
23 ≤ x < 25 (overweight)	1
25 ≤ x < 30 (obesity)	0.949 (0.798–1.128)
≥30 (extreme obesity)	1.172 (0.807–1.700)
*P*-value	0.0354

Mode: age, sex, smoking, drinking, exercise, income, education, metabolic syndrome, stress, and frequency of toothbrushing adjusted.
